# Divergent Changes in Bacterial Functionality as Affected by Root-Zone Ecological Restoration in an Aged Peach Orchard

**DOI:** 10.3390/microorganisms10112127

**Published:** 2022-10-27

**Authors:** Na Sun, Weiwei Zhang, Shangqiang Liao, Hong Li

**Affiliations:** 1 Institute of Plant Nutrition, Resources and Environment, Beijing Academy of Agriculture and Forestry Sciences, Beijing 100097, China; 2 Institute of Grassland, Flowers and Ecology, Beijing Academy of Agriculture and Forestry Sciences, Beijing Academy of Agriculture and Forestry Sciences, Beijing 100097, China

**Keywords:** soil property, alpha diversity, soil conditioner, orchard ecosystem, bacterial community

## Abstract

Soil restoration is a crucial approach to improving plant productivity in orchards with soil degradation, yield reduction, and fruit quality declination in China. A self-invented root-zone ecological restoration practice (RERP) with soil conditioner, or organic fertilizer, was employed in a degraded peach orchard in Beijing in 2020 to investigate the consequent impacts on soil bacterial composition and functionality at soil depths of 0–20 cm and 20–40 cm. Bacterial diversity was sensitive to RERP, especially in subsurface soil. RERP with soil conditioner significantly increased bacterial diversity, and affected abundances of certain genera, such as a significantly increased amount of *Bacillus* in surface soil and *Blastococcus*, *Microvirga*, *Nocardioides,* and *Sphingomonas* in subsurface soil. It also significantly affected abundances of bacterial functions related to metabolism in subsurface soil, particularly those with low abundance such as decreased transcription abundance and increased amino acid metabolism abundance. Soil bacterial functions were observably affected by bacterial diversity and composition, particularly in the deep soil layer. RERP affected bacterial functionality via responses of soil bacteria and bacteria-mediated alterations to the changed soil property. Correlation analysis between soil properties, bacterial taxonomy, and bacterial functions revealed that RERP affected bacterial functionality by altering the soil microenvironment with ample nutrients and water supply in root zone. Consequently, shifted bacterial functionality could have a potential in orchard ecosystem services in view of fruit yield and quality. Taken together, RERP had notably positive impacts on soil bacterial diversity and functions, and a prospect of increased plant productivity in the degrade orchard ecosystem.

## 1. Introduction

Fruit industry has rapidly developed and greatly contributed to increases in agricultural production efficiency, farmers’ income, and greening in rural areas in China [1]. Aged orchards with low productivity take up 40% of the total cropped area in China, proposing an urgent demand for soil restoration in degraded orchard ecosystem [2]. Continuous over-application of mineral fertilizer triggered soil degradations, such as soil acidification, structural deterioration, unstable soil microecosystem, declined microbial diversity, and severe soil-borne diseases, which was usually the case in aged orchards. Soil degradations directly affect plant ecological potential, plant productivity, and consequently the sustainability of orchard ecosystem [3,4]. A well-functioning and healthy orchard ecosystem is crucial to its eco-services, fruit production in particular.

In addition to replanting, soil restoration is another important practical measure to promote plant productivity in degraded orchards. Soil restoration practices generally focus on improving soil physical property, balancing soil nutrients, and regulating microbial community [3]. In the past decades, various soil restoration measures, such as mulching, irrigation, weeding, and application of bioorganic fertilizer or soil amendment, have been employed in different types of orchards [5,6,7,8,9]. However, these approaches failed to establish a stable soil environment with improved soil physicochemical properties. Due to the unique spatial distribution (amount and direction) of fruit tree roots, the nutrient uptake by 25% roots could meet the demand for plant growth [10]. This was evidenced by promoted soil ecosystem from partial roots regulation that often focused on physical, chemical, or microbial properties of root zone soil, but seldomly on all three aspects at the same time [6,11,12,13,14].

Orchard soil harbors a complex ecosystem with a diverse microbial community that can be affected by tillage, microbicide application, soil fumigation, and fertilization [6,8,15,16]. The interactions between soil bacterial diversity and agricultural practices in the orchard ecosystem have been studied in the last decade. For instance, herbicides application for weed control reduced microbial biomass and activity in the North Carolina Sandhill peach orchard [9]. Both irrigation amount and regimes were effective in influencing soil bacterial community in a peach orchard [9,17]. Bioorganic fertilizer application, mulching practice, and straw returning were reported with promoting effects on bacterial community and plant productivity in an apple orchard [5,7,8,18]. Root zone microorganisms in association with diseases from continuous cropping were also investigated in the peach orchard in California [19]. In recent years, a growing focus has been transferred to functional traits of soil microbial community [20,21,22]. However, research on bacterial functionality has become more complex due to the coexistence of annual growth cycle and life cycle of the perennial fruit trees in orchard ecosystem. To date, functional profiles of orchard ecosystem remain less characterized [23].

In this study, regulation of partial roots (root zone ecological restoration practice, RERP) was employed with a comprehensive improvement in soil physical, chemical, and microbial properties in a degraded peach orchard. The objectives of this study were to (1) characterize taxonomic structure and functional traits of soil bacterial community as affected by RERP; (2) reveal the associations between altered soil properties and bacterial diversity, composition, and functionality; and (3) address the interactions among bacterial taxonomy, bacterial functionality, and soil properties in an aged peach orchard. Findings of this study could provide theoretical basis and scientific support for proper managements in degraded orchards, and could contribute to understanding the mechanism of shifted soil bacterial functionality with partial roots regulation.

## 2. Materials and Methods

This study was conducted in a peach orchard in Yanqing District, Beijing since 2020. The peach orchard was constructed on a meadow cinnamon soil in spring 2008, taking up an area of 20 ha. Yanqing District has a warm, temperate, continental monsoon climate with an average annual temperature of approximately 8.5 °C and an average annual precipitation of approximately 443 mm. The experiment was conducted on an early-maturing peach cultivar named Chunxue that was planted in 2012. The row spacing and line spacing were 4 m and 3 m, respectively. Soil properties at depths of 0–20 cm and 20–40 cm before treatment are in Table 1.

### 2.1. Experimental Design

There were three treatments in this study. Treatment 1 (T1) was RERP with soil conditioner. A trench (12 m long, 0.8 m wide and 0.6 m deep) was dug 1.2 m away from the trees on their west side. The dug-out soil was placed in three separate piles and filled back to the trench according to soil depths (0–20 cm, 20–40 cm and 40–60 cm). In the meantime, a mixture of soil conditioner (3 t ha^−1^), organic fertilizer (15 t ha^−1^ DW), and mineral fertilizer (N:P_2_O_5_:K_2_O = 15:5:10, 900 kg ha^−1^) were equally split in triplicate and applied at soil depths of 20, 40, and 60 cm, separately. Treatment 2 (T2) was RERP with organic fertilizer. The applied materials and methods were the same as in T1, only without the application of 3 t ha^−1^ soil conditioner. The last treatment was the conventional practice (CK) in the orchard, including 650 kg ha^−1^ of urea (46%), 600 kg ha^−1^ of calcium superphosphate, and 310 kg ha^−1^ potassium sulfate each year. Total amount of mineral fertilizer of CK was evenly split into two applications in May and November with a radial furrow fertilization. The applied organic fertilizer was composted from sheep manure and maize straw (Beijing Beilangzhong Organic Fertilizer Factory, Beijing, China), with a 47.6% organic matter content and a total nutrient content (N, P_2_O_5_ and K_2_O) of 5%.

A total of 45 trees with the same growth conditions were picked out for the experiment. Each treatment had three replications, with five trees in each replication. Pest control and regular management of the peach tree were conducted as required, following the local practices for all treatments.

### 2.2. Soil Property Determination

Soil samples were taken on 19 July 2021 at depths of 0–20 cm and 20–40 cm for the determination of soil properties, including soil bulk density (SBD), soil field water-holding capacity (SFC), soil water content (SWC), soil porosity (SP), and contents of soil organic carbon (SOM), alkali-hydrolysable nitrogen (AN), available phosphorus (AP), available potassium (AK), water soluble calcium (WCa), water soluble magnesium (WMg), and available zinc (AZn). Each replication contained a mixed soil from three sampling sites (1.5 m apart from west side of the tree). SBD was determined by cutting ring method with sample rings of 100 cm^3^ volume [24]. SFC was determined using the cutting rings, and was calculated by the equation below:

SFC=(Ws−Wd)/Wd

where *Ws* is full saturated soil weight and *Wd* is dry soil weight [25]. SP was calculated by the equation below:

SP=(1−SBD)/Sg

where *Sg* is specific gravity [26].

SWC was calculated by the equation below:

SWC=(Wf−Wd)/Wd

where *Wf* is fresh soil weight and *Wd* is dry soil weight [27]. SOM was determined following the industry standard NY/T 85-1988 [28]. AN content was determined using alkali-diffusion method [29], while AK was extracted with ammonium acetate and then determined with a flame photometer [30]. WCa and WMg contents were determined with an atomic absorption spectrometry according to the industry standard NY/T 3242-2018 [31]. AZn was extracted with diethylenetriamine pentaacetic acid and then determined by atomic absorption method according to industry standard NY/T 890-2004 [32].

### 2.3. DNA Extraction, PCR Amplification and High-Throughput Sequencing

Total bacterial community genomic DNA was extracted from 0.5 g fresh soil samples (taken on 19 July 2021) at soil depths of 0–20 cm and 20–40 cm using the FastDNA Spin Kit (MP Biomedicals, Santa Ana, CA, USA). Quality of the extracted DNA was checked on 1.0% agarose gel, and the DNA concentration and purity were determined using a Nano Drop ND-1000 UV-vis Spectrophotometer (Thermo Scientific, Rockwood, TN, USA). A pair of primers 338F (5′-ACTCCTACGGGAGGCAGCAG-3′) and 806R (5′-GGACTACHVGGGTWTCTAAT-3′) was used for bacterial 16S rDNA gene amplification in the hypervariable V3-V4 region. The polymerase chain reaction (PCR) was conducted with an ABI GeneAmp^®^ 9700 PCR thermocycler (ABI, Los Angeles, CA, USA), with amplification conditions as follow: 3 min of initial denaturation at 95 °C, followed by 27 cycles of denaturing at 95 °C for 30 s, annealing at 55 °C for 30 s and extension at 72 °C for 45 s, and a final extension of 10 min at 72 °C. The PCR reaction mixture contained 4 μL of 5 × TransStart FastPfu buffer, 2 μL of 2.5 mM dNTPs, 0.8 μL of forward primer (5 μM), 0.8 μL of reverse primer (5 μM), 0.4 μL of TransStart FastPfu DNA Polymerase, 0.2 μL of BSA, 10 ng template DNA, and ddH_2_O addition up to 20 μL. Each sample was replicated three times in the amplification and mixed up afterwards. PCR products were then purified using the AxyPrep DNA Gel Extraction Kit (Axygen Biosciences, Union City, CA, USA) and quantified using Quantus™ Fluorometer (Promega, Madison, WI, USA). Purified amplicons were pooled in equimolar, and paired-end were sequenced on an Illumina MiSeq PE300 platform (Illumina, San Diego, CA, USA) according to the standard protocols by Majorbio Bio-Pharm Technology Co. Ltd. (Shanghai, China). The obtained gene sequences from Illumina platform are now deposited in the National Center for Biotechnology Information (NCBI) Sequence Read Archive with an accession number PRJNA835607.

The sequencing data were processed by fastp (https://github.com/OpenGene/fastp, Version 0.20.0, accessed on 25 August 2022, HaploX Biotechnology, Shenzhen, China) and FLASH (http://www.cbcb.umd.edu/software/flash, version 1.2.7, accessed on 25 August 2022, University of Maryland, College Park, MD, USA) for barcode and primer trimming. Low-quality reads of less than Q20 were removed [33,34]. The 18 test samples had a total number of 958,788 effective sequences with an average length of 417 bp. High quality sequences were clustered into operational taxonomic units (OTUs) with a 97% similarity using UPARSE (http://drive5.com/uparse/, Version 7.1, accessed on 25 August 2022, Robert Edgar, Tiburon, CA, USA). RDP classifier (http://rdp.cme.msu.edu/, Version 2.2, accessed on 25 August 2022, Michigan State University, East Lancing, MI, USA) was used for comparison of the representative sequence of each OTU to the database Silva (Release115, http://www.arb-silva.de, accessed on 25 August 2022, Marine Microbiology and Jacobs University, Bremen, Germany) with a confidence threshold of 0.7 [35].

### 2.4. Data Analysis

Alpha-diversity metrics were assessed for diversity (Shannon and Simpson) and richness (Chao1 and ACE) using Mothur (http://www.mothur.org/, Version 1.31.2, accessed on 25 August 2022). Analysis of variance (ANOVA) was conducted for alpha diversity estimates, soil properties, and functional profiles as a function of three treatments at different soil depths using the PROC ANOVA procedure in the SAS statistical software package (SAS Institute Inc., Cary, NC, USA). Means were compared using the Least Significant Difference (LSD) test at the 5% probability level. PICRUSt2 (Phylogenetic Investigation Communities by Reconstruction of Unobserved States) was used to predict functionality of bacterial community. Bacterial community composition of all treatments was compared via stacked column diagram analysis in Origin 2021 (Origin Lab Corporation, Northampton, MA, USA). Beta-diversity (genus level) among all treatments were calculated with the Bray-Curtis dissimilarity metric and visualized in a Principal Coordinates Analysis (PCoA) plot using R version 3.3.1 (Linux-GNU). The relationships between soil characteristics and alpha-diversity estimates were analyzed using PROC CORR and PROC REG in SAS. Spearman correlation analysis was conducted between soil characteristics and bacterial community composition, soil characteristics, and bacterial community functionality, alpha diversity estimates and bacterial functionality, and bacterial community composition and functionality. A redundancy analysis (RDA) was performed for relationships between soil characteristics and bacterial community functionality in CANOCO 5.0. The manual forward-selection procedure was used to determine significance of soil characteristic variables (*p* < 0.05) using a Monte Carlo test with 499 permutations.

## 3. Results

### 3.1. Soil Properties Affected by RERP

RERP significantly influenced soil properties in 2021, with a more pronounced positive effect of T1 than T2 (Table 2). Decreases of SP, moisture (SFC and SWC), and nutrients (AN, AP, AK, WCa, WMg, and AZn) with increasing soil depth was observed in all treatments. T1 improved soil property by increasing permeability (decreased SBD and increased SP), soil moisture (SFC and SWC), soil nutrients (AN, AK, WCa, WMg and AZn), and SOM contents at both soil depths. T2 had a lesser positive impact on soil properties with few responded aspects (SOM, SFC, AK, and AZn contents) that mainly in surface soil.

### 3.2. Alpha Diversity of Soil Bacterial Community Affected by RERP

RERP had significantly increased diversity and richness of bacterial communities in comparison to CK (Table 3). Sobs, Shannon, ACE, and Chao1 were significantly higher in T1 (0–40 cm) and T2 (0–20 cm) than CK, indicating a thorough improvement of RERP with soil conditioner that extended to subsurface soil. Furthermore, a decrease in bacterial diversity with increasing soil depth was generally observed for CK and T2.

### 3.3. Beta Diversity of Soil Bacterial Community Affected by RERP

The shared OTU number of all three treatments decreased from 3794 in surface soil to 3319 in subsurface soil (Figure S1). The number of OTU that exclusive to T1 (1133–1550) were higher than that of CK and T2, indicating a positive effect of RERP with soil conditioner on soil bacterial diversity, especially in subsurface soil. Shared OTU number between T1 and T2 was higher than that of CK with T1 or T2, suggesting a profound effect of RERP on alpha diversity of bacterial community in the peach orchard.

**Figure 1:**
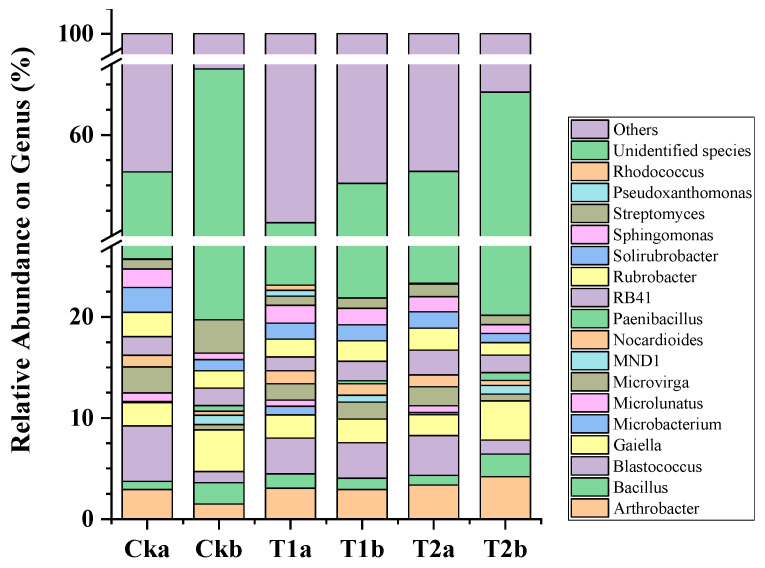
Soil bacterial community composition on genus level.

Thirty-one genera (relative abundance >1% in at least one sample) accounted for 51.31–56.40% and 55.22–66.53% of the total bacterial sequences in surface and subsurface soil, separately (Figure 1). Soil depth significantly affected bacterial abundance. The relative abundances of *Blastococcus*, *Microvirga*, *Nocardioides*, *Solirubrobacter* and *Sphingomonas* were higher in surface soil, while *Bacillus*, *Gaiella* and *Streptomyces* abundances were more enriched in subsurface soil for CK and T2. T1 had a profound effect bacterial community, with a tendency of bringing bacterial abundances at two soil depths to the same level. For instance, *Bacillus* abundance was significantly increased in surface soil, but significantly decreased in subsurface soil. *Blastococcus* and *Microvirga* abundances were enriched in subsurface soil but decreased in surface soil. However, 16 and 18 out of the 31 genera were unidentified (named as “unclassified” or “norank”) in top and deep soil layers, separately. As the majority of bacterial community, unidentified or abundance <1% genera remained a challenging reservoir in soil of the orchard ecosystem, especially in deeper layers (which acctounted for 83.44% of the total sequences).

**Figure 2:**
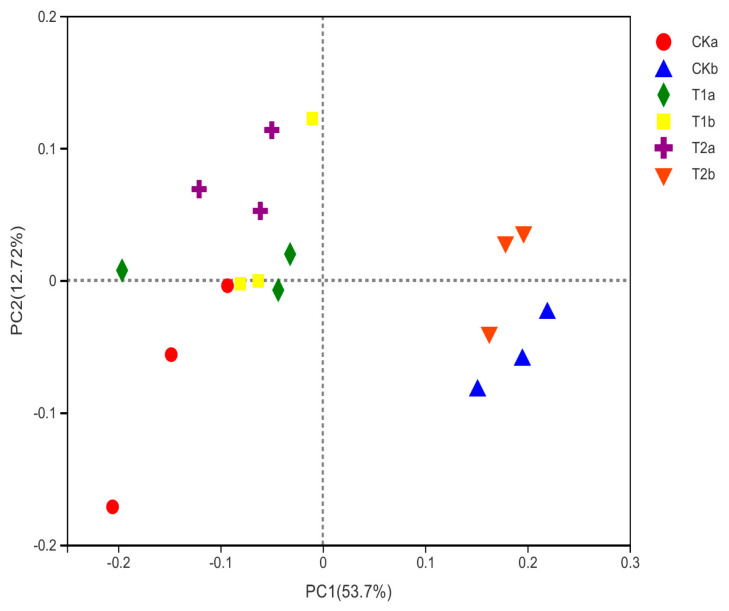
Principle coordinate analysis of soil bacterial community on genus level.

Principal coordinate analysis revealed that the first two components explained 66.4% (PC1 of 53.7 % and PC2 of 12.7%) of the total variance of bacterial communities (Figure 2). Bacterial community of T1 was clearly differentiated from CK and T2 in subsurface soil, indicating a prominent effect of RERP with soil conditioner in deep soil layer. Bacterial community of surface soil samples demonstrated variations in PC2, with CK separated from T1 and T2.

### 3.4. Soil Bacterial Functionality

Bacterial functional profiles of three treatments were predicted using PICRUST2 at different soil depths (Table 4 and Table 5). “Metabolism”, “genetic information processing”, and “environmental information processing” were the most abundant functions in biochemical metabolic pathways, with relative abundances of 52.67, 15.52, and 14.31%, respectively (Table 4). RERP significantly affected the relative abundances of “metabolism”, “genetic information processing”, and “organismal systems”. “Genetic information processing” was more abundant in subsurface soil for all three treatments, while “metabolism” and “organismal systems” were more abundant in surface soil for CK and T2. Compared to CK, T1 significantly reduced abundance of “metabolism” in surface soil, and increased abundances of “metabolism” and “organismal systems” in subsurface soil. RERP with soil conditioner significantly changed the abundance of potential functions of bacterial community in the root zone of peach orchard, especially in subsurface soil.

Ultimately, 23 out of the 41 sub-functions of soil bacterial community had relative abundances >1%. “Membrane transport”, “amino acid metabolism”, “carbohydrate metabolism”, “replication and repair”, and “energy metabolism” were the most abundant functions. Soil depth and RERP significantly affected relative abundance of 13 sub-functions, such as “amino acid metabolism”, “transcription”, “translation”, “metabolism”, “metabolism of other amino acids”, and “signaling molecules and interaction” (Table 5). All of these sub-functions had higher relative abundances in surface soil than in subsurface soil for CK and T2, except for “transcription” and “translation”. T1 was effective in affecting sub-function abundances in subsurface soil, especially the ones with low abundance. T1 had decreased “transcription” abundance, and increased abundances of “amino acid metabolism”, “metabolism”, and “signaling molecules and interaction” when compared to CK.

## 4. Discussion

### 4.1. Soil Bacterial Diversity as Affected by RERP

**Figure 3:**
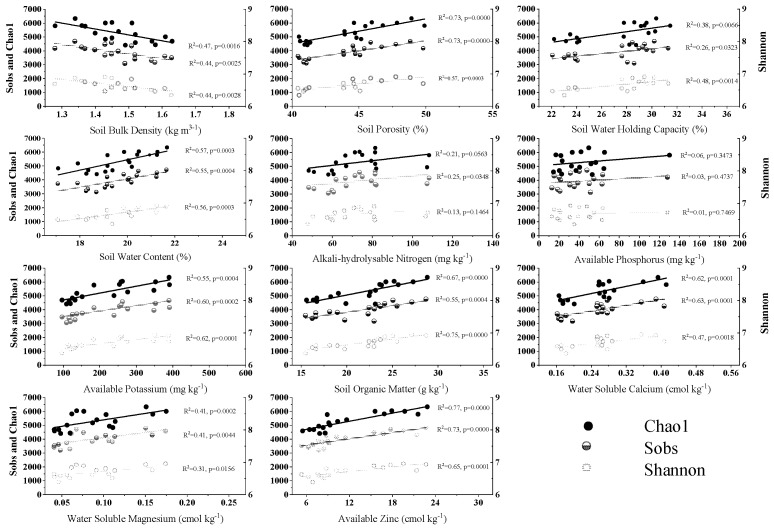
Correlation analysis between soil characteristics and alpha diversity. Left Y axis indicates Sobs and Chao1 values, while right Y axis indicates Shannon value.

Soil bacteria is highly sensitive to the surrounding environment. It has a quick response on its taxonomical structure, and which is thereby often adopted as an indicator of ecosystem health [36,37]. RERP affected soil bacterial alpha diversity via the altered soil physicochemical properties, evidenced with their strong correlations (Figure 3). Alpha diversity estimates (Sobs, Shannon, and Chao1) were generally linearly correlated to soil physical properties (SBD, SP, SFC, and SWC), AK, SOM, and micro elements (Ca, Mg and Zn), but were only mildly correlated to AN and AP. We concluded three possible causes as follows: (1) the trench digging in RERP had a similar effect to deep plough, which improved soil properties by decreasing SBD, increasing SP and SWC and consequently bacterial diversity in the root zone [38]. Appropriate SWC could stimulate microbial reproduction, accelerate microbial decomposition, and consequently increase soil nutrient availability [39,40]. (2) The organic fertilizer addition in RERP increased nutrient availability and SOM content, resulting in increased bacterial diversity. Similarly, bacterial diversity was reported significantly correlated with various soil characteristics after organic amendments addition in previous studies, such as SOM, AN, AP, and AK contents in an apple orchard [6], SOM and total N contents in wheat-maize rotation [41], and soil EC, AN, total N, total P, and total K contents in urban forest [42]. (3) RERP with soil conditioner applied mineral substrate (mainly montmorillonite), super absorbent polymers and micro elements, thereby increased soil water retention capacity and contents of AK, Ca, Mg, and Zn. This may have alleviated the imbalanced soil nutrient situation in the aged orchard [43], which was supported by the significant linear correlations between bacterial diversity and WCa, WMg, and AZn contents. However, a previous study reported a contrary conclusion where high Ca and Mg contents had negative impacts on soil microbial diversity in an apple orchard [44], potentially due to different ecosystems or the high background value of these elements that may exceeded their thresholds in that study.

**Figure 4:**
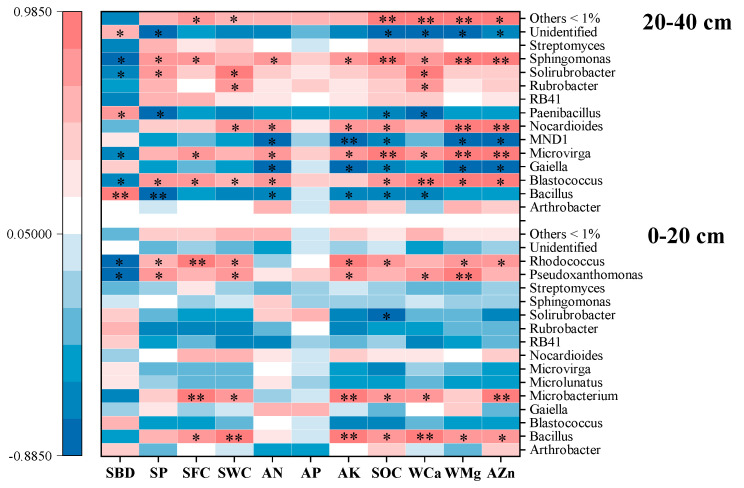
Analysis of soil bacterial community composition as affected by soil property. Spearman correlation analysis between soil characteristics and bacterial abundance on genus level. * indicates significant correlation (*p* < 0.05), while ** indicates extremely significant correlation (*p* < 0.01).

Spearman correlation analysis revealed significant correlations between soil property and bacterial genera abundances that have been affected by RERP (Figure 4). Four genera, *Blastococcus*, *Bacillus*, *Sphingomonas,* and *Solirubrobacter* abundances were in positive correlations with soil AN. Wang et al. [45] reported similar results of increased relative abundances of *Blastococcus* and *Bacillus* in soil with high soil total N and available N contents resulting from a 13-year cover crop (white clover) practice. *Bacillus* and *Blastococcus* degrade organic material through production proteolysis [46,47], which was beneficial to the accumulation of SOM and soil nutrients. SBD was in negative correlation with these four genera. Decreased SBD from RERP had increased soil porosity and water content, which might be the possible cause for the enrichment of these four genera [39,40,48].

The above results suggested that bacterial diversity can be purposively altered by agricultural practices. For example, RERP in this study had a pronounced positive effect on bacterial community that have been lasted for two years after application in the peach orchard. Compared to previous agricultural approaches, RERP realized a comprehensive improvement of soil property on chemical, physical, and biological aspects. In the meantime, the in situ RERP is relatively simple and convenient to perform than replanting. Further investigation on whether this RERP-regulated soil bacterial community could reach to a new balance should be addressed.

### 4.2. The structure Soil Bacterial Functionality as Affected by RERP

Microbial metabolic functions rather than just taxonomic composition were key to many questions in ecosystem ecology and biogeochemistry [37]. Structure of biochemical metabolic pathway functions (>1%) changed over location, environmental conditions, and human interferences in this and previous studies [23,49,50,51]. “Metabolism” had a relative abundance of 52.67% (averaged over all samples) in this study, which was far higher than it (26.42%) in the *Pinus sylvestris* ecosystem in Hulun Buir sandy soil [49], and lower than it in maize and alfalfa ecosystem in loess plateau soil (69.20 and 70.22%, respectively) [50]. Interestingly, relative abundance of “metabolism” was comparable to that in a mango orchard (51.73 and 52.36%, respectively) [23]. Similar results were also observed for functions “genetic information processing” and “environmental information processing” [23,49,50]. However, greater discrepancies have shown on number and abundance of sub-function. Agricultural ecosystems with human interference had more sub-functions than natural ecosystem. For instance, *Pinus sylvestris* ecosystem had 31 sub-functions [49], which was less than maize and alfalfa ecosystem (46 sub-functions) [50], mango orchard ecosystem (43 sub-functions) [23], and peach orchard ecosystem (42 sub-functions) in this study. Meanwhile, the abundances of sub-functions were affected by anthropogenic disturbance of the experimental ecosystem. Greater variances on abundances of “membrane transport”, “amino acid metabolism” and “carbohydrate metabolism” were found between *Pinus sylvestris* and peach orchard ecosystems, rather than mango and peach orchard ecosystems [23,49].

Due to the infertile soil and cold climate, *Pinus sylvestris* grown in Hulun Buir had a priority in harnessing nutrient and energy from the surrounding environment to ensure sustainability of the ecosystem, which reflected in functions of “environmental information processing” and “membrane transport” [49]. Maize and alfalfa that grew in the rainfed area on loess plateau were under scarcity of soil water, thereby enriched sub-functions of “carbohydrate metabolism”, “amino acid metabolism”, “energy metabolism”, and “metabolism of cofactors and vitamins” for carbohydrates, amino acids, energy, and vitamins to survive the extreme environment [50]. Despite discrepancies of location, bacterial diversity, and taxonomy, the peach orchard in this study had a similar functional structure with the mango orchard [23]. This was possibly related to the characteristics of the orchard ecosystem: perennial trees with frequent human disturbance (fertilization, irrigation, spraying pesticide, and pruning) for higher economic return. As concluded by Yan et al. [52], plant exerts selections on bacterial taxonomy based on their functionality via abiotic environmental factors within the existing ecosystem.

### 4.3. Soil Bacterial Functionality as Affected by Soil Physicochemical Properties

Soil microbes encode metabolic pathways that drive elemental cycles in most ecosystems, which then shaped earth’s surface chemistry over billions of years [53]. Yet, our knowledge is still in its infancy of the coupling mechanism between microbial communities and abiotic physicochemical processes. Soil bacterial functionality can be affected by both biotic and abiotic factors, while soil type and crop specie are decisive in whichever one becomes the limiting factor [54]. Therefore, it is necessary to investigate the response of orchard soil bacterial functionality to environmental disturbances, in view of responsive patterns, magnitude, and specific soil characteristics and bacterial functions.

**Figure 5:**
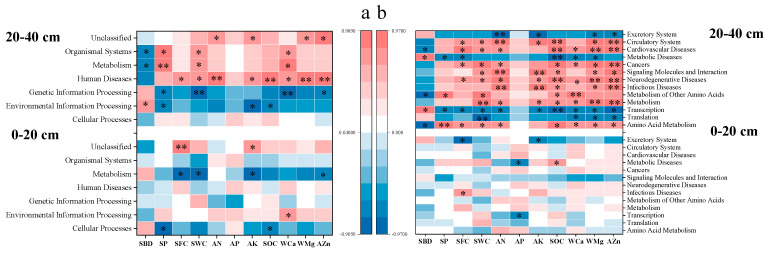

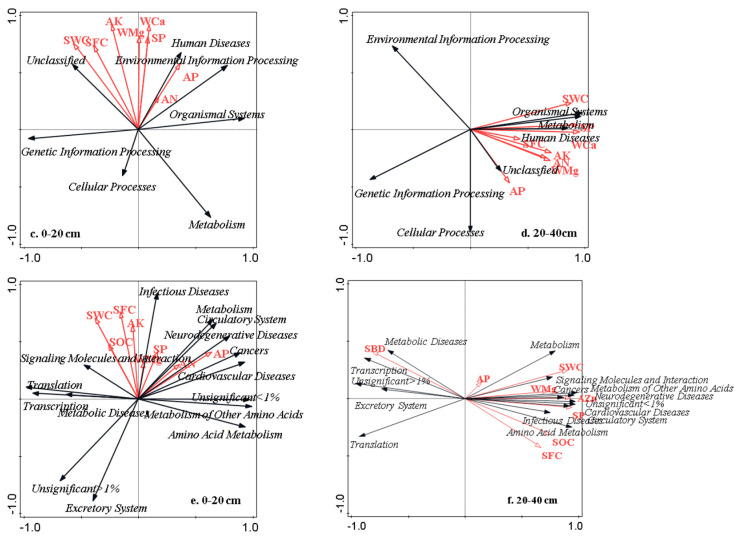
Analysis of soil bacterial functions as affected by soil property. Spearman correlation analysis between soil characteristics and functions in biochemical metabolic pathways (**a**); and sub-functions (**b**). Redundancy analysis of the relationship between soil characteristics and functionality (biochemical metabolic pathways, (**c**,**d**); sub-functions, (**e**,**f**). * indicates significant correlation (*p* < 0.05), while ** indicates extremely significant correlation (*p* < 0.01).

According to spearman correlation analysis, relative abundance of function “metabolism” was negatively correlated with SFC, SWC, AK, and AZn (*p* < 0.05) in surface soil (Figure 5a). In subsurface soil, “metabolism” abundance was in positive relation to SP, SWC, and WCa, and in negative relation to SBD. The function “genetic information processing” abundance was negatively correlated to SP, WCa, and AZn in subsurface soil. Soil property was in strong correlations with sub-functions in subsurface soil rather than in surface soil (Figure 5b). Sub-functions related to metabolism and signaling molecules were significantly correlated with SFC, SWC, AN, SOM, WCa, WMg, and AZn in this study. A previous study [55] has reported similar findings of soil total organic C in positive correlations with “membrane transport” and “amino acid metabolism”. Moreover, the addition of Ca, Mg, and Zn from soil conditioner resulted in their significant positive correlations with functions related to metabolism (including “amino acid metabolism”, “metabolism”, and “metabolism of other amino acids”), which were relevant to plant productivity including fruit yield and quality. This is in accordance with previous studies, where N, P, Ca, and Mg application increased growth and yield of field-grown tomato [56], and ratios of N/Ca, K/Ca and (Mg + K)/Ca were in significant correlation with fruit quality [57]. Balanced macro- and micro-nutrients from poultry manure application increased soil fertility, plant growth, and fruit yield of pineapples [58]. Zhang et al. [59] reported pomelo yield increased by 34.2 and 26.8% in 2018 and 2019, respectively, from lime + Mg addition.

Soil water (SWC or SFC) and SP were determined as the main contributors for the variations of bacterial functions (biochemical metabolic pathways and sub-functions; Figure 5c–f). This indicated that RERP affected bacterial functionality mainly through the alteration of soil moisture and compactness, which happened to be the limiting factors for aged orchard soil in arid and semi-arid regions [3,4].

The soil bacterial community function was sensitive to abiotic factors in the studied orchard (particularly the subsurface layer), which were consistent with the distribution of fruit tree roots with obvious hierarchical and concentrated stratification characteristics [4]. On the other hand, growth zone of peach root was mainly located in deep soil layer with stable environmental conditions during summer, which was consistent to the situation in this study. This is the likely the cause for more pronounced effects of RERP in subsurface soil than in surface soil. Furthermore, it is noticed that balanced soil nutrients with micronutrient additions in RERP is beneficial to improve bacterial community functions related to metabolism, particularly “amino acid metabolism”. However, effects of RERP on soil may attenuate over time. Seasonal shifts on soil property affecting bacterial metabolic functioning were also reported [48]. Therefore, deep knowledge on how long this RERP effect could last, and if it is seasonal dependent in needed. This could provide information for temporal regulation of bacterial functions at different growth stage in peach orchards.

### 4.4. Soil Bacterial Functionality as Affected by Alpha and Beta Diversities

Major biogeochemical reactions are driven by a limited set of energy-transducing metabolic pathways that involves with a variety of microbes [37]. Impacts of bacterial taxonomy on ecosystem function have been intensively investigated [60,61], with a focus on dynamic associations among soil C, taxonomic structure, and functional profile of soil microbial community in recent years [62,63,64]. Higher alpha and beta diversities of bacterial community could further impact soil multifunctionality, as indicated by their positive correlations in natural ecosystem [22].

**Figure 6:**
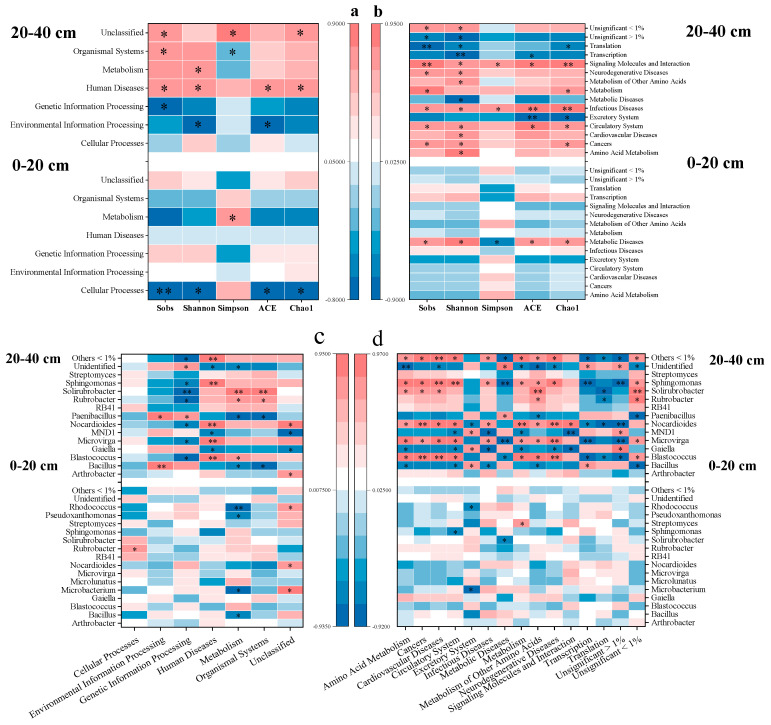
Spearman correlation analysis between taxonomy and functionality of bacterial community. Alpha diversities in relation to functions in biochemical metabolic pathways (**a**) and sub-functions (**b**); genus abundance and functions in biochemical metabolic pathways (**c**) and sub-functions (**d**). * indicates significant correlation (*p* < 0.05), while ** indicates extremely significant correlation (*p* < 0.01).

According to spearman correlation analysis, the correlations between functions in biochemical metabolic pathways and alpha diversity estimates were stronger in subsurface soil than in surface soil (Figure 6a). Alpha diversity estimates were negatively correlated with “genetic information processing” and “organismal systems” (*p* < 0.05), but positively correlated with “metabolism” (*p* < 0.05) in subsurface soil. Alpha diversity correlations with sub-functions were also more profound in subsurface soil, as exhibited by stronger correlations with diverse sub-functions (Figure 6b). A previous study reported similar results which emphasized the potential of a diverse microbial community in improving agricultural ecosystem functionality [36].

Understanding of the role of bacterial composition in influencing the bacterial community assembly process and functional traits is essential for a sustainable ecosystem [54]. Functions in biochemical metabolic pathways were mildly correlated with soil bacteria in surface soil (Figure 6c), with “cellular processes” positively correlating to *Rubrobacter* abundance, and “metabolism” in negative correlation with *Bacillus*, *Microbacterium*, *Pseudoxanthomonas,* and *Rhodococcus* abundances. Strong correlations between bacterial abundances and diverse functions were observed in subsurface soil. Generally, functions of “environmental information processing”, “genetic information processing”, “metabolism”, and “organismal systems” were significantly correlated to genera *Blastococcus*, *Rubrobacter*, *Sphingomonas*, *Nocardioides*, *Bacillus*, *Microlunatus,* and *Pseudoxanthomonas* abundances. We also analyzed the relationships between bacteria and 13 sub-functions related to metabolism and discovered that their strong correlations in subsurface soil (Figure 6d). For instance, “amino acid metabolism”, “metabolism”, and “metabolism of other amino acid” were in significant correlations with *Blastococcus*, *Microvirga*, *Sphingomonas*, *Nocardioides Rubrobacter,* and *Bacillus* abundances in the deep soil layer.

All these bacterial genera significantly affected by RERP were involved in nutrients (such as C, N, P, K, and S) cycling, which could be related to their correlated metabolic functions. For example, *Bacillus* and *Blastococcus* degrade organic material through production proteolysis [46,47], which were beneficial to the accumulation of SOM. *Rubrobacter* was reported involving in the process of dark oxidation of sulfur compounds, which then affected sulfur availability for peach plants [65]. *Sphingomonas* had extensive metabolic ability to degrade many aromatic compounds and the certain species of this genus could synthesize valuable extracellular biopolymers [66]. *Nocardioides* were relevant to C and N nutrients for plant growth [67]. *Microlunatus*, a novel actinobacterium, contributed to the decomposition of organic matter [68,69]. *Pseudoxanthomonas* represents a relatively newly characterized group of gamma-proteobacterium, which can enhance essential nutrients cycling and consequently soil fertility [70,71,72].

The results of this study indicated extensive functional redundancy in soil bacterial community at regional ecosystem scale, with distinct bacteria encoding the same metabolic functions. This also suggests the concurrence of cooperation and competition of bacteria in the same ecosystem [73]. Louca et al. [37] reported similar conclusion of multiple distinct microorganisms performing similar metabolic functions on local scale within nine metabolic functional groups for all bromeliads in east coast Brazil. Another interesting finding was the strong correlations between bacterial functionality (sub-functions in particular) and bacteria in subsurface soil, indicating that regulation of root zone bacterial community should be in deeper soil layer for high plant productivity. Understanding the shifts of bacterial taxonomy following different agricultural management practices at different soil depths is important for selecting suitable strategies to maintain the functional diversity of ecosystems. In the meantime, we did not look into rare genera (<1%), which may have played an over-proportional role in biological processes and being the major driver for bacterial community multifunctionality [36]. Further investigation on the related aspects could be take looked into in the future.

## 5. Conclusions

The results of this study indicated that RERP increased soil bacterial diversity in the aged peach orchard, especially deep soil layer (20–40 cm). Compared to conventional practice, RERP altered soil bacterial composition, especially certain genera related to soil nutrient cycling that enriched in deep soil layer. Significant shifts of functional profile (mainly with increased sub-functions in subsurface soil) as affected by RERP were observed at both soil depths (0–40 cm). Soil physicochemical properties and the selected metabolic functions were decisive in stabilized systemic characteristics in the open microbial system in this study. Bacterial functions related to metabolism, particularly “amino acid metabolism”, were significantly affected by balanced soil nutrients after micronutrients addition in RERP. In summary, RERP improved soil property with balanced nutrients, decreased bulk density, and increased water holding capacity, thereby establishing a bacterial community with increased diversity, and altering bacterial taxonomy and metabolic functions (especially sub-functions) in subsurface soil.

Although the mechanism and effectiveness of bacterial functionality in improving plant productivity remain unclear, this study delivered valuable information for better understanding of the complex processes of bacterial multifunction in degraded orchard ecosystem. Due to the seasonal shifts of bacterial diversity, composition, and functionality, we advocate further investigation on their correlations with biological processes and ecological services of orchard ecosystem. New evaluation indicators and systems should be proposed to further explain anthropogenic activity on soil bacterial diversity and ecological service function in orchard ecosystem.

## Figures and Tables

**Table 1:** Soil property of the peach orchard at soil depth of 0–20 cm and 20–40 cm before treatment in 2020.

Soil Depth	Alkali-Hydrolysable N	Available P	Available K	Total N	Total P	Total K	SOM	WCa	WMg
cm	mg kg^−1^			%			g kg^−1^	cmol kg^−1^	
0–20	105	81.9	457.67	0.17	0.1	2.03	22.8	0.31	0.12
20–40	90.47	19.77	133.33	0.11	0.06	1.98	18.9	0.24	0.06

N, nitrogen; P, phosphorus; K, potassium; SOM, soil organic matter; WCa, water soluble calcium; WMg, water soluble magnesium.

**Table 2:** Soil property of all three treatments at soil depth of 0–20 cm (a) and 20–40 (b) in 2021.

Treatment	SBD	SP	SOM	SFC	SWC	Alkali-Hydrolysable N	Available P	Available K	WCa	WMg	AZn
	g cm^3−1^	%	g kg^−1^	%		mg kg^−1^			cmol kg^−1^		
CKa	1.47 ^a,b^	44.64 ^b^	18.02 ^d^	23.24 ^c^	18.40 ^c^	81.69 ^a^	52.41 ^a^	138.05 ^c^	0.27 ^b^	0.10 ^b^	8.40 ^d^
CKb	1.57 ^a^	41.01 ^c^	16.21 ^d^	23.81 ^c^	18.94 ^b,c^	53.04 ^c^	23.97 ^c^	115.02 ^c^	0.18 ^c^	0.04 ^d^	6.31 ^e^
T1a	1.35 ^c^	48.89 ^a^	27.25 ^a^	30.46 ^a^	21.40 ^a^	81.43 ^a^	41.39 ^a,b^	378.04 ^a^	0.39 ^a^	0.16 ^a^	21.40 ^a^
T1b	1.44 ^b,c^	45.80 ^b^	24.37 ^b^	28.60 ^b^	20.26 ^a,b^	73.33 ^b^	29.38 ^b,c^	258.21 ^b^	0.27 ^b^	0.07 ^c^	16.80 ^b^
T2a	1.41 ^b,c^	44.70 ^b^	23.18 ^b,c^	29.71 ^a^	19.77 ^b,c^	69.35 ^b^	33.94 ^b,c^	269.78 ^b^	0.27 ^b^	0.10 ^b^	10.30 ^c^
T2b	1.56 ^a^	40.87 ^c^	21.83 ^c^	28.24 ^b^	18.72 ^c^	61.90 ^b,c^	27.58 ^b,c^	155.91 ^c^	0.18 ^c^	0.06 ^c,d^	8.60 ^c,d^

Numbers followed by different letters are significantly different (*p* < 0.05). SBD, Soil Bulk Density; SP, Soil Porosity; SOM, Soil Organic Matter; SFC, Soil Field Water Holding Capacity; SWC, Soil Water Content; WCa, water soluble calcium; WMg, water soluble magnesium; AZn, available zinc.

**Table 3:** Alpha diversity estimates of bacterial community at soil depth of 0–20 cm (a) and 20–40 cm (b) of three treatments in 2021.

Treatment		Diversity		Richness	
	Sobs	Shannon	Simpson	ACE	Chao1
Cka	3698 ^c^	6.52 ^b^	0.0067 ^a^	4922 ^c^	4982 ^c^
CKb	3375 ^d^	6.46 ^b^	0.0038 ^c^	4953 ^c^	4655 ^c^
T1a	4423 ^a^	6.82 ^a^	0.0043 ^b,c^	6058 ^a^	6045 ^a^
T1b	4385 ^a^	6.82 ^a^	0.0042 ^b,c^	5935 ^a,b^	5956 ^a^
T2a	4044 ^b^	6.71 ^a^	0.0049 ^b,c^	5459 ^b,c^	5477 ^b^
T2b	3263 ^d^	6.50 ^b^	0.0053 ^a,b^	5106 ^c^	4620 ^c^

Numbers followed by different letters are significantly different (*p* < 0.05).

**Table 4:** Functional abundance in biochemical metabolic pathways of bacterial community of each treatment.

Treatment	Metabolism	Genetic Information Processing	Cellular Processes	Environmental Information Processing	Human Diseases	Organismal Systems	Unclassified
Cka	52.951 ^a^	15.292	3.639	14.249	0.767 ^a,b^	0.824 ^a^	12.278
CKb	52.457 ^c^	15.759	3.601	14.420	0.710 ^c^	0.797 ^b,c^	12.255
T1a	52.674 ^b^	15.379	3.534	14.410	0.798 ^a^	0.820 ^a^	12.384
T1b	52.769 ^a,b^	15.498	3.613	14.162	0.780 ^a^	0.819 ^a^	12.357
T2a	52.766 ^a,b^	15.453	3.641	14.148	0.782 ^a^	0.816 ^a,b^	12.394
T2b	52.350 ^c^	15.768	3.620	14.418	0.727 ^b,c^	0.789 ^c^	12.329

Numbers followed by different letters are significantly different (*p* < 0.05).

**Table 5:** Relative abundances of sub-functions of bacterial community of each treatment.

Treatment	Amino Acid Metabolism	Translation	Transcription	Metabolism	Metabolism of Other Amino Acids	Infectious Diseases	Neurodegenerative Diseases	Signaling Molecules and Interaction	Cancers	Metabolic Diseases	Cardiovascular Diseases	Circulatory System	Excretory System
Cka	11.254 ^a^	4.080 ^b^	2.405 ^c^	2.352 ^a,b^	1.975 ^a^	0.314 ^a,b^	0.204 ^a,b^	0.201 ^a^	0.106 ^a^	0.082 ^b^	0.012 ^a,b^	0.021 ^a,b^	0.0445 ^a,b^
CKb	11.091 ^c^	4.257 ^a^	2.530 ^a^	2.289 ^c^	1.878 ^b^	0.304 ^b^	0.176 ^c^	0.190 ^b,c^	0.086 ^b^	0.085 ^a^	0.007 ^c^	0.017 ^c^	0.0453 ^a^
T1a	11.223 ^a^	4.088 ^b^	2.433 ^b,c^	2.367 ^a^	1.966 ^a^	0.321 ^a^	0.219 ^a^	0.196 ^a,b^	0.113 ^a^	0.083 ^a,b^	0.013 ^a^	0.022 ^a^	0.0436 ^b^
T1b	11.201 ^a,b^	4.139 ^a,b^	2.444 ^b,c^	2.353 ^a,b^	1.945 ^a,b^	0.321 ^a^	0.206 ^a,b^	0.200 ^a^	0.106 ^a^	0.083 ^a,b^	0.011 ^a,b^	0.021 ^a^	0.0440 ^b^
T2a	11.194 ^a,b^	4.124 ^b^	2.439 ^b,c^	2.364 ^a^	1.952 ^a^	0.324 ^a^	0.209 ^a,b^	0.202 ^a^	0.106 ^a^	0.082 ^b^	0.011 ^a,b^	0.022 ^a^	0.0436 ^b^
T2b	11.119 ^b,c^	4.261 ^a^	2.497 ^a,b^	2.308 ^b,c^	1.884 ^b^	0.309 ^b^	0.185 ^b,c^	0.190 ^c^	0.0907 ^b^	0.084 ^a^	0.008 ^b,c^	0.018 ^b,c^	0.0452 ^a^

Numbers followed by different letters are significantly different (*p* < 0.05).

## Data Availability

The obtained gene sequences from Illumina platform are now deposited in the National Center for Biotechnology Information (NCBI) Sequence Read Archive with an accession number PRJNA835607.

## Supplementary Materials

The following supporting information can be downloaded at: https://www.mdpi.com/article/10.3390/microorganisms10112127/s1, Figure S1: Venn diagram analysis of soil bacterial community in surface soil (0–20 cm, **a**) and subsurface soil (20–40 cm, **b**) on OTU level.
