# Supplementary material for: Divergent Changes in Bacterial Functionality as Affected by Root-Zone Ecological Restoration in an Aged Peach Orchard
Source: Microorganisms. 2022 Oct 27;10(11):2127. doi: 10.3390/microorganisms10112127 (PMC9699486; doi:10.3390/microorganisms10112127)
Supplement: Supplementary file 1 [file microorganisms-10-02127-s001.zip › microorganisms-1958794-supplementary.pdf]

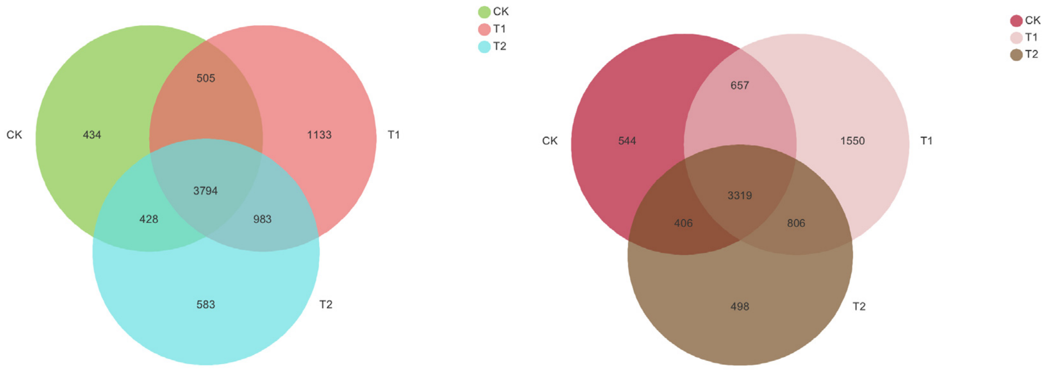

Figure S1 Venn diagram analysis of soil bacterial community in surface soil (0-20 cm, a) and subsurface soil (20-40 cm, b) on OTU level
